# Anorexia nervosa and the size-weight illusion: No evidence of impaired visual-haptic object integration

**DOI:** 10.1371/journal.pone.0237421

**Published:** 2020-08-27

**Authors:** Manja M. Engel, Karlien van Denderen, Anne-Richtje Bakker, Andrew W. Corcoran, Anouk Keizer, H. Chris Dijkerman

**Affiliations:** 1 Utrecht University, Faculty of Social and Behavioural Sciences, Experimental Psychology/Helmholtz Institute, Utrecht, The Netherlands; 2 Leontienhuis, Zevenhuizen, The Netherlands; 3 Cognition and Philosophy Laboratory, Monash University, Melbourne, Victoria, Australia; Anglia Ruskin University, UNITED KINGDOM

## Abstract

Body image disorders in anorexia nervosa (AN) patients and recovered AN (RAN) patients have been suggested to stem from aberrant integration of sensory information. Previous research by Case et al. (2012) used the size-weight illusion (SWI) to study multisensory integration in AN. Their results showed a diminished SWI in AN patients, which they interpreted as evidence of decreased integration of visual and proprioceptive information. However, their method did not distinguish between visual and haptic size information, which was presented concurrently while making weight judgements. Therefore, the reported effect might be attributed to integrating visual, haptic size cues, or a combination of both processes with proprioceptive input. Here, we use the SWI to investigate the integration of visual and haptic object-related sensory information in a sample of AN patients (*n* = 30), RAN patients (*n* = 29) and healthy controls (HC) (*n* = 29). We aimed to distinguish the contribution of visual and haptic object size by including separate visual and haptic SWI conditions. In addition to explicit measures, we included grip force measurements to assess implicit expectations about object weight. We further analysed the correlation between the SWI and a visual body size estimation (VSE) task. In contrast to Case et al. (2012), we found no evidence of differential SWI experience between groups. All participants reported a stronger visual SWI compared to haptic SWI. Grip force rate (but not peak) showed evidence of motor adaptation for the larger object in the visual condition. Furthermore, there was no correlation between the VSE and SWI, indicating no relation between perceived object weight and body size estimation. These results do not support the hypothesised impairment of visual-haptic object related integration in AN.

## Introduction

Anorexia nervosa (AN) is an eating disorder where patients have an intense fear of gaining weight, despite being (severely) underweight. In addition, patients display a distorted body image that is not only limited to distorted thoughts about the body but also involves disturbances in body perception [1–4]. These body image disturbances (BID) are strongly associated with the development and maintenance of eating disorders (ED), and relapse is known to be predicted by the severity of the disorder [5–7]. BID symptoms are still present in recovered ED patients [3, 8]. This persistence of symptoms reflects the complexity of the disorder and highlights the importance of research to BID.

BID in AN involves negative bodily cognitions (thoughts) and attitudes (affect), but also involves disturbances in various perceptual modalities suggesting an oversized mental body representation in AN [2, 8–11]. BID symptoms regarding these perceptual distortions are suggested to be caused by impairments in multisensory integration [12, 13]. The exact mechanism underlying impairments in multisensory integration in AN is not yet fully understood.

Multisensory integration is often measured by perceptual illusions, where the outcome of the illusion is informative regarding how the brain prioritizes or integrates certain sensory information. The *size-weight illusion* (SWI), also known as the Charpentier illusion, is a classic example of such a perceptual illusion [14]. This illusion occurs when a smaller object is perceived as heavier than a larger object that has the same mass. A proposed cause of the illusion is a violation of the expectation that larger objects are heavier. The illusion arises when the discrepancy between actual and expected sensory feedback leads people to perceive the opposite of their prior expectation [15]. However, the SWI is unlikely to be explained by sensorimotor mismatch alone. When measuring grip- and load force during the SWI, Flanagan and Beltzner [16] showed that motor adaptation occurs after repeated lifting, where anticipated grip- and load force were precisely scaled to the object weight. Interestingly, this motor-adaptation does not affect the experience of the illusion [17]. Density explanations could also account for the SWI; denser objects are known to be heavier in weight [18, 19] and participants might actually base their judgements on the increased density of the smaller object rather than its weight. Although there is, as of yet, no single satisfying explanation, researchers agree that when the threshold is too low for humans to differentiate between two weights, the smaller weight will be perceived as heavier [e.g. 20].

To our knowledge, so far only one study has investigated the SWI in patients with AN. Case et al. [21] measured multisensory integration in AN patients through a SWI battery. In each trial participants were presented with one small and one large disk in the palm of each hand, and asked to report the weight of a small disk relative to a larger disk while looking at them. The participants were always presented with the same small disk (90g) together with a large disk. The large disks were manipulated in weight (90-210g), but did not vary in size. The results showed a diminished SWI in AN compared to healthy controls (HC); i.e. unlike HC, AN patients were less likely to perceive the smaller disk as heavier when the actual weight of the large and small disks was similar, despite normal weight discrimination abilities [21]. Case et al. [21] attributed the apparently diminished SWI in AN to a decreased integration of visual information (because the SWI is strongly modulated by visual size cues) and proprioceptive information.

Although this conclusion is interesting there are a few methodological points in this study that need to be addressed. First, the sample size Case et al. [21] used was rather small (AN: *n* = 10; HC: *n* = 10); consequently, the finding could be spurious (significant effects in low-powered studies are more likely to be unreliable [22]). Second, their design did not enable the role of visual and haptic information to be disentangled: Participants were allowed to see and feel the size of the objects at the same time while making their weight judgements, therefore the reported effect could be due to visual size processing, proprioception, or some combination thereof. As the authors point out, their results are unable to discriminate between a number of possible explanations for why patients experience a diminished illusion; visuo-proprioceptive differences, visuo-tactile-proprioceptive differences, visuo-tactile differences, or differences in visual processing alone.

In this study, we set out to replicate Case et al.’s [21] findings in a larger sample, and to isolate whether differences in the SWI between AN and HC are modality-specific. To this end, we devised a paradigm involving separate visual and haptic SWI conditions. In addition to an explicit (verbal report) measure, we included implicit (grip force) measures of the SWI. In line with previous work, we expected the explicit measure to show the perceived object weight and the implicit measure to show the anticipated grip force scaled to the actual object weight [16, 17]. We use both measures to investigate weight perception and implicit expectation of object weight differences between groups.

Furthermore, we included a group of recovered anorexia (RAN) patients within the study. Previous research has shown that perceptual BID symptoms are still present in this group, persisting after otherwise successful treatment [3, 8]. Engel and Keizer [8] showed disturbances in visual size perception and affordance perception, but not in bodily attitudes in patients who completed ED treatment.

In their paper, Case et al [21] speculated that the visuo-proprioceptive integration deficit might be related to overestimation of one’s own body size in AN. We therefore also included a visual body size estimation task to explore the link between multisensory integration (as indexed by the SWI) and body size estimation.

In sum, this study used a SWI battery to distinguish between illusory effects in the visual and haptic modalities in a sample of AN patients, RAN patients, and HC participants. We obtained explicit (verbal response) and implicit (grip force) measures. Furthermore, we analysed correlations between the SWI and a visual body size estimation task. This study was designed to investigate multimodal integration and has an exploratory nature, hence specific hypotheses will not be stated regarding the differences in grip force. For the explicit measures on the SWI we expected to find differences between AN and HC consistent with the previous work by Case et al. [21]. In line with our own findings on body image tasks in RAN patients we expected that AN and RAN patients would show larger overestimations compared to HC on the visual body size estimation task [8].

## Method

### Ethics statement

The current study was approved by the Faculty Ethics Review Board of Utrecht University, Faculty of Social and Behavioural Sciences. The study adhered to the tenets of the Declaration of Helsinki [23]. Each participant was informed about the study; they received oral and written information on the purpose and procedure prior to the experiment. All participants signed an informed consent form before taking part in the study.

### Participants

AN and RAN patients were recruited from the Leontienhuis, Stichting JIJ, and GGZ Rivierduinen Eetstoornissen Ursula, these are patient organisations and mental health institutions in The Netherlands. HC were volunteers of the Leontienhuis and undergraduate students from Utrecht University. Undergraduate students received course credit for participation. Participants were included in the AN and RAN group if they had a current or past diagnosis of AN or eating disorder not otherwise specified (EDNOS), as symptoms are similar in EDNOS [24]. Diagnostic criteria for AN in past or present were checked with the SCID IV-I, part H in participants. Inclusion criteria for HC were no past or present ED. Participants were excluded if they had any known neurological disorder or psychotic illness.

Eighty-nine individuals were recruited who fitted our inclusion criteria. Data from one participant who did not experience the SWI (responded that the objects were identical in weight) were excluded from analysis. Our final sample consisted of 30 AN patients, 29 RAN patients, and 29 HC. Of this sample, grip force data were missing for 3 RAN patients due to technical error (equipment failure). Data for one AN and one RAN patient were not collected in the SWI haptic condition because the participants felt uncomfortable wearing a blindfold. One HC participant did not participate in the VSE.

All participants were females above the age of 18 with a mean age of 29.10 (*SD* = 10.04). No significant age difference was found between groups, *F*(2,87) = .478, *p* = .622. No significant handedness difference was found between groups, *F*(2,87) = .721, *p* = .489. BMI was significantly different between groups, *F*(2,80) = 16.468, *p* < .001. Post-hoc analysis with Tukey correction showed that BMI in AN patients was significantly lower than in RAN patients (*p* < .001) and HC (*p* < .001). No differences in BMI were found between RAN patients and HC (*p =* .572). See Table 1 for demographical and clinical information per group.

**Table 1:** Demographics and clinical assessment.

	HC	RAN	AN
	*n* = 29	*n* = 29	*n* = 30
**Demographics**			
Age	28.8 ± 10.9	30.4 ± 10.9	27.53 ± 8.1
BMI	22.2 ± 3.45	21.4 ± 2.4	18.0 ± 2.5^a^
Right—Handedness	96.6% (29)	85.2% (23)	86.7% (26)
**Diagnosis**			
AN	--	20	21
EDNOS	--	9	9
No ED	30	--	--
Age ED diagnosis	--	17.9 ± 6.4	20.7 ± 6.6
Duration treatment (yrs)	--	3.4 ± 3.0	2.8 ± 3.2
Time since treatment completion (yrs)	--	4.5 ± 6.0	--

^*a*^ *p* < .001

### Materials

#### Structured clinical interview for DSM-IV Axis I, part H

The SCID IV-I is a semi structured clinical interview. Part H (Eating Disorders) was used to check the diagnostic criteria for the presence of an acute or past ED in participants.

#### Size weight illusion

The weights used were black cylinders and consisted of a small (A, 252 grams, 5 cm diameter) and large (C, 505 grams, 7 cm diameter) control weight, and a small (X, 370 grams, 5 cm diameter) and large (Z, 373 grams, 7 cm diameter) experimental weight. The control set looked identical to the experimental set. Practice weight B had a diameter of 6 cm and a weight of 378 grams.

**Fig 1:**
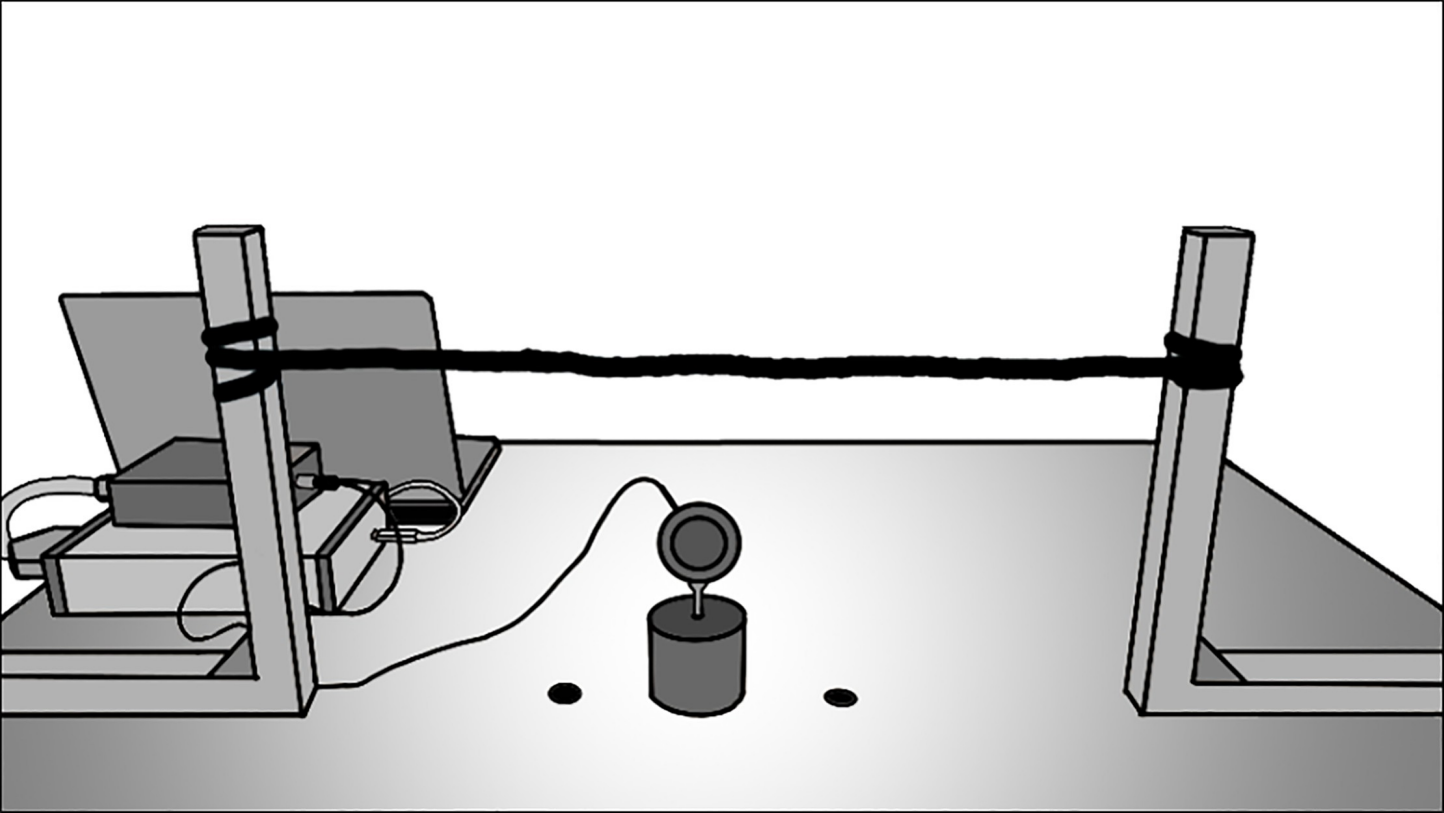
Set up of size weight illusion. Participants placed their hands on the black markers. Participants lifted the object by the handle in both conditions until it reached the ribbon. Here they held the object steady for 5 seconds. In the visual condition participants were only allowed to look at the object before lifting it by the handle. In the haptic condition participants were blindfolded and were allowed to feel the object with their left hand. They were then asked to place the left hand back on the black marker and lift the object with their right hand by the handle.

Two vertical aluminium stands were attached to a desk, the space between stands was 50 cm. Three markers were placed between the stands at 20, 25, and 30 cm to indicate starting points for weights and hand. A line was drawn on both aluminium stands to indicate 5 cm from table, and a blue ribbon was tied horizontally between both stands as a tactile indicator for the hands when the weights were lifted 5 cm, see Fig 1.

#### Grip force

Grip force was measured by a removable stainless steel handle mounted on the top of the weights by a steel clip, minimizing object sway. The handle was equipped with a six-axis force/torque sensor (Mini40-E Transducer with Strain Relieved Cable, ATI Industrial Automation) that measured the forces and torques applied by the digits in six dimensions. Sandpaper with 180 grain was glued to the handle to prevent slippage.

#### Visual size estimation

Arrow shaped stickers were used for indicators of body width. A calliper was used to measure actual width of body parts.

### Procedure

After signing the informed consent, participants filled out a questionnaire with questions concerning demographic and clinical information. Next, the SCID IV-I part H was administered after which participants took part in both visual and haptic condition of the SWI. In both conditions, participants were asked to place their hands on the outer markers on the table (Fig 1). A practice weight (B) was placed on the centre marker. Participants were allowed to practice holding the grip force handle and lifting the practice object vertically and steadily to a height of 5 cm. The vertical line was used to check if participants lifted the object to a height of 5 cm when their hand reached the ribbon (the height of the ribbon was adjusted if needed). There was no fixed amount of practice trials, the experiment started when participants showed good understanding of the procedure. Both the visual and haptic condition started with the control set (A(small) and C(large)) with 7 lifts (ACACACA) and was immediately followed by the experimental set (X(small) and Z(large)) with 13 lifts (XZXZXZXZXZXZX). The control condition allowed participants to learn the relationship between size and weight. Changing objects was done out of sight from the participant.

**Fig 2:**
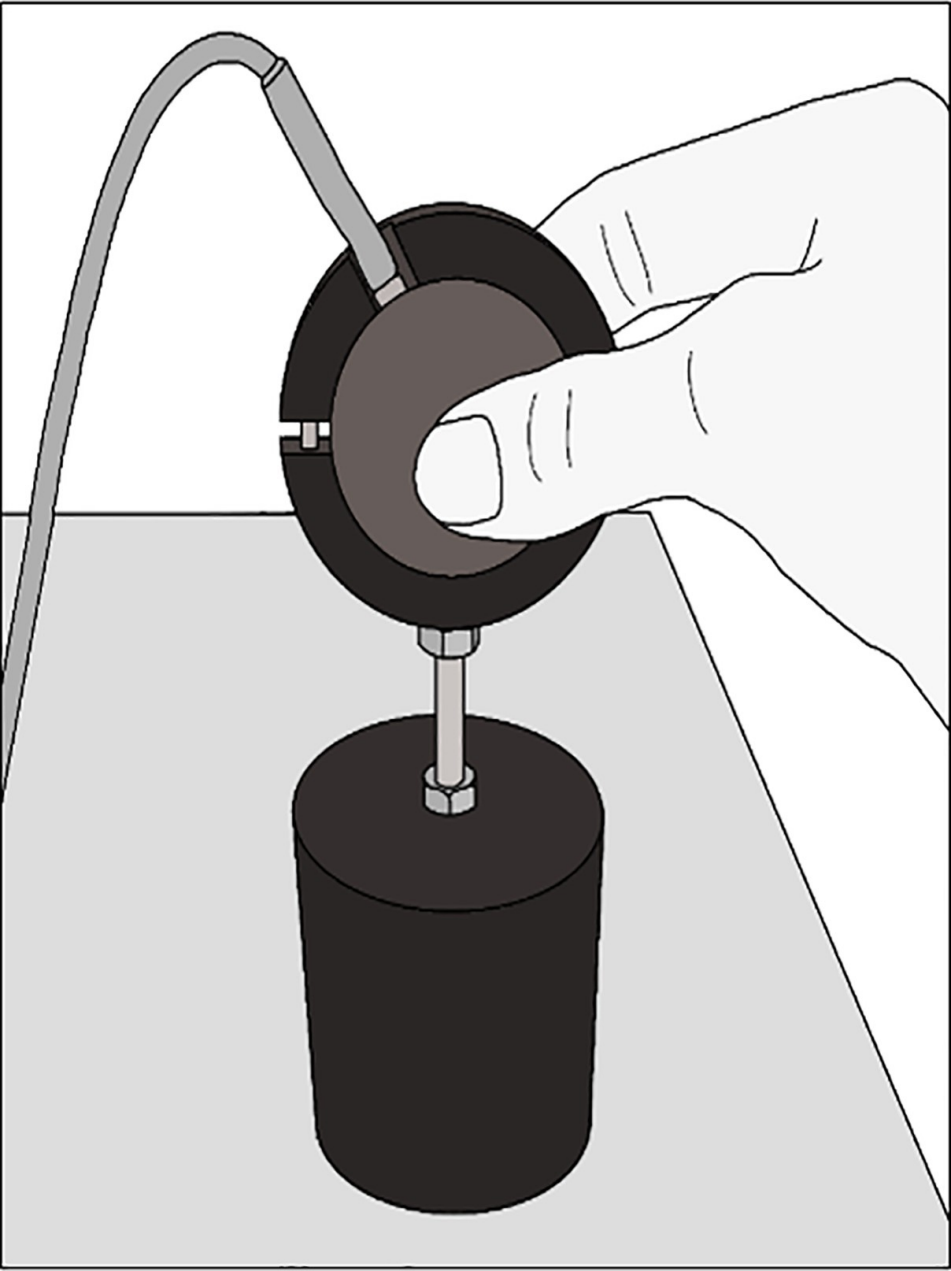
Grasping of the transducer. Participants placed their index finger and thumb on the handle. The handle measured grip force and was attached to each object by an iron pin to reduce sway.

After hearing a beep, participants lifted the object by placing their right thumb and index finger on the centre of the grip force handle (Fig 2) and lifting it 5 cm in a vertical direction, until the hand reached the ribbon. The object was held steady for approximately 5 seconds. After putting the object down, the participants answered the following question: *“Is this object heavier than the previous*?*”* (a forced choice routine was used) with an exception for the first object lifted.

In the visual condition participants were allowed to look at (but not touch) the object before and during lifting. The object was lifted by the handle with the participant’s right hand. In the haptic condition a blindfold was used to prevent participants from seeing the object. Here participants were allowed to feel the shape of the object with their left hand without lifting the object, for 5 seconds. After hearing a beep the participant lifted the object with the right hand, to a height where they felt the ribbon (5 cm) and held it steady for approximately 5 seconds. The question *“Is this object heavier than the previous*?*”* was asked after each lift. The order of the visual and haptic condition was counterbalanced over subjects.

Next, the Visual Size Estimation (VSE) task was administered. Participants stood in front of a wall, at a distance of 1 meter, facing the wall. An arrow shaped sticker was placed on the wall and participants placed a second sticker with their right hand on the wall, estimating the width of a body part. The space between stickers indicated the estimated width. Points of interest were shoulders, waist, and hips. Order of estimations was counterbalanced over participants. The right sticker was always removed between estimations to prevent comparison of estimation. At the end of the experiment, the actual size of shoulders, waist and hips of the participants were measured by the experimenter.

### Statistical analysis

For the SWI the percentage of the verbal weight judgement responses was calculated after each set of trials. In the control condition this percentage reflects the proportion of trials in which the heavier object was correctly identified (perceiving the object as heavier when it is actually heavier). In the experimental condition the percentage reflects the amount of responses in line with the SWI (perceiving the smaller object as heavier than the larger object even though they are approximately the same weight). ANOVAs were conducted in IBM SPSS Statistics for Windows, Version 24 [25] with group as the between-subjects independent variable. Dependent variables for the ANOVA were the percentages of answers for the control condition and the experimental condition.

Grip force data were processed with Matlab R2018a (The MathWorks, Inc., Natick, MA, USA). Fz forces obtained by the Mini40-E Transducer were used to calculate grip force peaks and grip force rates. The force signals were processed with a finite impulse response moving average filter (filter.m MATLAB function, windowsize = 5). For each trial, the peak grip force (N) was determined with the findpeaks.m function (with ‘MinPeakProminence’ of 50 and ‘MinPeakHeight’ of 500). For grip force rate, lift off was determined as the lowest point before the selected peak. Grip force rate was calculated using a two-point central difference equation, with the following formula:

$$\frac{df}{dx}|{}_{{x=x_{i}}^{2}}=\frac{f(x_{i}+1)-f(x_{i}-1)}{x_{i}+1-x_{i}-1}$$

Trials were removed when the lift was inaccurate (e.g. participant did not follow instructions or moved their index finger and thumb during lifting), or due to technical error. Average peak grip forces (N) and grip force rates (N/s) of each lift were calculated for each group per condition (visual, haptic) and size (small weight (X) and large weight (Z)) for the SWI trials only. To establish if force adaptation occurred over time, we divided the 12 lifting trials per visual and haptic condition into 2 consecutive blocks of 6. We then took the average of the grip force rate of the small and large object for each block and examined the data across all subjects. Two mixed ANOVAs were conducted in IBM SPSS Statistics for Windows, Version 24 [25] with group as the between-subjects independent variable, and condition, size, and lifts as within-group independent variables. For the first mixed ANOVA the grip force rate (N/s) was the dependent variable; grip force peak (N) was the dependent variable for the second mixed ANOVA.

For the VSE, the percentage of misestimation was calculated for each body part as follows:

$$100*\frac{estimation-actual\;size}{actual\;size}.$$

Three ANOVAs were conducted (one for each body part) in IBM SPSS Statistics for Windows, Version 24 [25] with percentage of misestimation as the dependent variable and group as the independent variable.

A Pearson correlation was conducted to test for a relation between the VSE and the visual SWI. The variables used were, percentage of misestimation of each body part (shoulders, hips and waist) and the percentage of answers in line with the visual SWI.

Assumption of normality was checked with the Shapiro-Wilk Test. Where this test indicated the violation of this assumption, the Kruskal-Wallis test was used to test for between-group differences. If this test was significant Mann-Whitney U tests were used as post hoc contrasts. The Wilcoxon Signed Ranks test was used to test for within-group differences. Levene’s test was used to assess homogeneity of variance. Where this test indicated heteroskedasticity, *Welch’s F* was computed [26].

Bonferroni correction was used for all post hoc comparisons for all ANOVAs. Missing data were handled by listwise deletion.

We checked for correlation of clinical variables (‘BMI’, ‘age of onset’, ‘treatment duration’, ‘time since completion of treatment’ as described in Table 1) with all SWI measurements and VSE measures. No significant correlations were found and these variables were therefore not used as covariates in our main analysis.

## Results

### SWI

The Kruskal-Wallis and the Wilcoxon Signed Ranks tests were used to compare medians between groups and modality, respectively. All participants responded equally accurately in the visual control condition and haptic control conditions.

The Kruskal-Wallis test showed no differences between groups in the visual SWI condition (*H*(2) = 1.315, *p* = 0.518). No significant difference was found in the haptic SWI between groups (*H*(2) = 2.453, *p* = .293). The Wilcoxon Signed Ranks test revealed that participants experienced a stronger visual than haptic SWI (*W*_*s*_ *=* 2784.5, *p* = .001). See Table 2 for medians and interquartile range.

**Table 2:** Median and interquartile range of SWI.

	Visual			Haptic		
	*Mdn* (*IQR*)			*Mdn* (*IQR*)		
	AN	RAN	HC	AN	RAN	HC
**Control**	100 (0.0)	100 (0.0)	100 (0.0)	100 (0.0)	100 (0.0)	100 (0.0)
**Exp**	91.67 (16.67)	91.67 (16.67)	91.67 (16.67)	87.50 (25.0)	83.33 (25.0)	83.33 (25.0)

In the control condition, percentages indicate the proportion of trials in which the heavier weight was correctly identified. In the experimental condition, percentages indicate the proportion of trials in which the smaller weight was rated heavier (i.e. consistent with the SWI).

### Grip force

**Fig 3:**
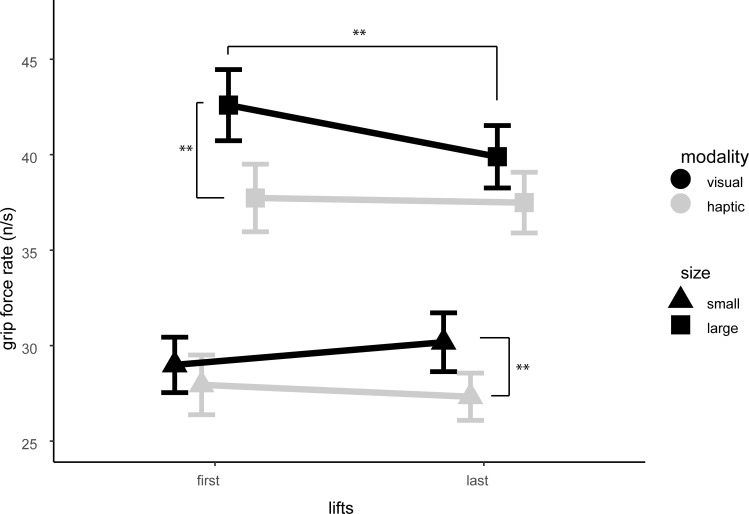
Condition*Size*Lift interaction. Participants show reduced grip force rate over lifts for the larger object in the visual condition.

A mixed ANOVA showed significant main effects of modality and size on grip force rate (N/s). A significant interaction effect was found for modality*size*lifts, see Fig 3. Post hocs revealed a significant reduction in grip force rate in the visual modality for the large object over lifts (*p* = .004). Participants showed higher grip force rates in the first set of lifts for the larger object in the visual modality compared to the haptic modality (*p* = .002). Higher grip force rates in the last set of lifts were found for the smaller object in the visual modality compared to the haptic modality (*p* = .009). No main effect for group was found, nor any interactions with group (see Tables 3 and 4 for statistics).

**Table 3:** Results of two mixed measures ANOVA for grip force peak rate (N/s) and grip force peak (N).

Source	*df*	*F*	*p*	*partial ղ^2^*
**Grip force rate (N/s)**				
**Condition**	**1, 82**	**7.192**	**.009**	**.08**
**Size**	**1, 82**	**328.369**	**< .001**	**.80**
Lifts	1, 82	1.082	.301	
Group	2, 82	2.505	.088	
Condition * Group	2, 82	.407	.667	
Size * Group	2, 82	2.562	.083	
Lifts * Group	2, 82	1.810	.170	
Condition * Size	1, 82	3.257	.075	
Condition * Lifts	1, 82	.119	.731	
Size * Lifts	1, 82	2.916	.091	
Condition * Size * Lifts	**1, 82**	**5.617**	**.020**	**.06**
Condition * Size * Group	2, 82	.370	.692	
Size * Lifts * Group	2, 82	.129	.879	
Condition * Lifts * Group	2, 82	.710	.495	
Condition * Size * Lifts * Group	2, 82	.991	.375	
**Grip force peak (N)**				
Condition	1, 82	.101	.752	
**Size**	**1, 82**	**270.991**	**< .001**	**.77**
Lifts	1, 82	3.616	.061	
Group	2, 82	1.551	.218	
Condition * Group	2, 82	.514	.600	
Size * Group	2, 82	2.897	.061	
Lifts * Group	2, 82	.108	.897	
Condition * Size	1, 82	1.057	.307	
Condition * Lifts	1, 82	2.176	.144	
Size * Lifts	1, 82	1.417	.237	
Size * Lifts * Group	2, 82	.595	.554	
Condition * Size * Group	2, 82	.367	.649	
Condition * Size * Lifts	1, 82	3.706	.058	
Condition * Lifts * Group	2, 82	.841	.435	
Condition * Size * Lifts * Group	2, 82	.455	.636	

**Table 4:** Means and standard deviations of grip force peak rate (N/s).

	HC	RAN	AN	Total
**Visual**	32.54 ± 11.47	38.80 ± 18.27	34.39 ± 11.60	35.01 ± 14.05
Small (X)	27.52 ± 11.03	32.59 ± 17.76	28.17 ± 09.86	29.32 ± 13.18
Large (Z)	37.56 ± 12.68	44.94 ± 19.71	40.62 ± 14.25	40.94 ± 15.80
**Haptic**	30.76 ± 10.76	36.08 ± 17.20	30.01 ± 10.24	32.20 ± 13.14
Small (X)	26.81 ± 10.16	31.19 ± 15.76	24.12 ± 08.90	27.28 ± 12.10
Large (Z)	34.71 ± 12.20	40.98 ± 19.11	35.91 ± 12.12	37.12 ± 14.83

**Fig 4:**
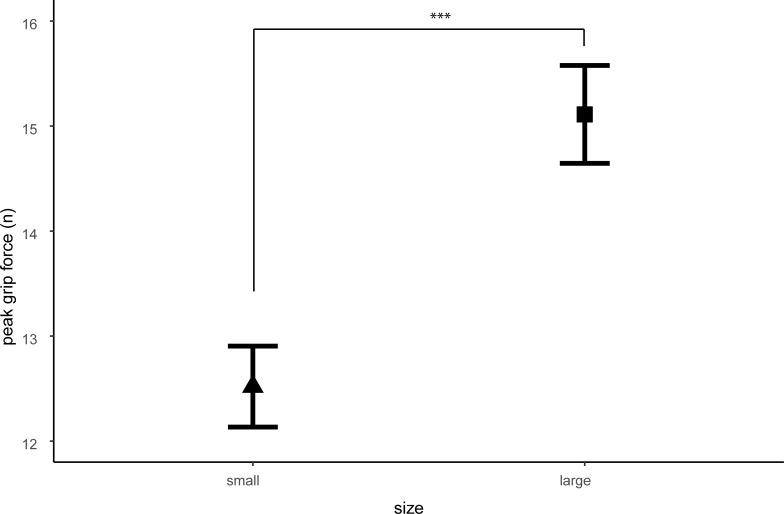
Main effect of size for grip force peak. Participants show higher peaks in grip force for the larger object.

A second mixed ANOVA showed a significant main effect of size on grip force peak (N), see Fig 4. No significant main effect for group was found, nor any interactions with group (see Tables 3 and 4 for statistics). These results indicate that all participants showed higher grip force peaks for the larger compared to the smaller object (*M*_*S*_ = 13.78, *SD*_*S*_ = 3.99; *M*_*L*_ = 15.00, *SD*_*L*_ = 4.34). No significant difference in grip force peak between the first and second block of trials for the visual and haptic modality were found, indicating no evidence of peak force adaptation over time. See Table 5 for means and standard deviations.

**Table 5:** Means and standard deviations of grip force peak (N).

	HC	RAN	AN	Total
**Visual**	13.27 ± 3.73	14.42 ± 4.66	13.52 ± 3.71	13.71 ± 4.02
Small (X)	12.11 ± 3.45	12.90 ± 4.33	12.06 ± 3.18	12.34 ± 3.64
Large (Z)	14.43 ± 4.15	15.88 ± 5.22	14.98 ± 4.35	15.08 ± 4.56
**Haptic**	13.37 ± 3.37	14.73 ± 5.40	13.11 ± 3.63	13.72 ± 4.21
Small (X)	12.45 ± 3.05	13.30 ± 5.15	11.81 ± 3.31	12.50 ± 3.92
Large (Z)	14.30 ± 3.95	16.17 ± 5.80	14.42 ± 4.10	14.93 ± 4.69

### VSE

**Fig 5:**
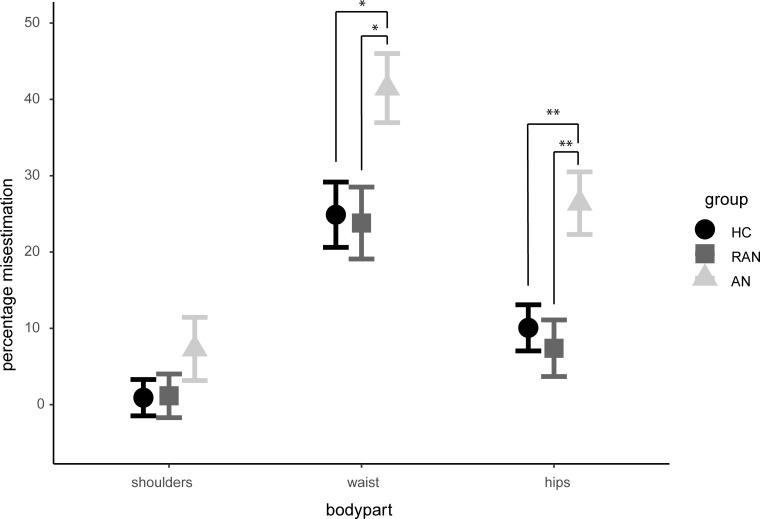
Visual size estimation between groups.

An ANOVA showed significant differences in estimation between groups for waist (*F*(2, 84) = 4.98, *p* = .009, *ω* = .10) and hips (*F*(2, 84) = 8.09, *p* < .001, *ω* = .17), see Fig 5. No differences were found between groups for shoulders, (*Welch’s F*(2, 54.33) = 1.03, *p* = .391). Post-hocs showed that AN patients made larger overestimations for waist size than RAN patients (*p* = .019, *d* = 0.7) and HC (*p* = .026, *d* = 0.7). No difference was found between RAN patients and HC (*p* = 0.985). This was also evident in the estimations for hips where AN patients made significantly larger estimations compared to RAN patients (*p* = .001, *d* = 0.9) and HC (*p* = .005, *d* = 0.8). No difference was found between RAN patients and HC (*p* = .868). See Table 6 for means and standard deviations.

**Table 6:** Percentage of misestimation of shoulder, waist, and hips of participants.

	HC	RAN	AN
**Shoulders**	0.92 ± 12.84	1.16 ± 14.86	7.31 ± 23.06
**Waist**	24.89 ± 23.05	23.80± 24.47	41.48 ± 25.19
**Hips**	10.07 ± 16.26	7.39 ± 19.09	26.39 ± 22.83

Pearson correlation tests were conducted to establish if associations exists between SWI measures and VSE measures. Correlation results indicated only one significant negative association between VSE hips estimates in AN and visual SWI condition (*r*(31) = -.404, *p* = .024). However, this result did not survive the Bonferroni correction (critical *p* = .004).

## Discussion

BID in AN is related to disturbances in sensory integration. Previous research by Case et al. [21] studied visuo-tactile-proprioceptive integration in AN using a SWI paradigm. They attributed their findings of a diminished SWI in AN to a decreased integration of visual and proprioceptive information. However, Case et al. [21] used a version of the SWI that presented visual size and haptic size cues simultaneously. Hence, it is unclear whether the differences they found derived from visual or haptic information processing, or the integration of these processing streams. In our study we additionally tested the visual and haptic SWI separately, thereby allowing comparison between integration within and across modalities. Our sample consisted of AN patients, RAN patients and HC. We measured the strength of the SWI through verbal report (explicit) and grip force. It is assumed that peak grip force (N) and grip force rate (N/s) reflect implicit expectations of object weight [27]. In addition, we checked for correlations between the verbal report of the SWI (object size related) and a visual body size estimation task (body size related) to establish whether there was a relation between object weight estimations and body size estimations, respectively.

Results showed that all participants experienced a stronger visual than haptic SWI. In contradiction to the findings of Case et al. [21], we found no difference in the strength of the SWI between AN and HC participants. This observation implies no evidence of visual-haptic integration deficit in AN (and additionally, RAN) patients compared to HC. Similarly, we found no significant between-group differences on either of our grip force measures. Grip force rate did reveal an interaction between condition, size and lift: grip force rate was reduced in the later lifts of the larger object in the visual condition. This decrease in grip force rate is indicative of motor adaptation, which is in line with previous research [16]. Interestingly we did not find any such effect in the haptic condition. Results further revealed a main effect of size for grip force peak, where participants showed a higher peak for the larger object compared to the smaller object. We did not find any evidence of motor adaptation in the grip force peak. Results of the visual body size estimation task revealed an overestimation for the width of waist and hips, but not for shoulders, in AN patients compared to RAN and HC. No differences were found for the estimation of shoulder width between all groups. Furthermore, our correlation analysis did not reveal a relation between the visual body size estimation task and the SWI.

In contrast to the previous results found by Case et al. [21], we did not find a difference in the SWI between our clinical groups and HC. Case et al. [21] reported a diminished SWI in AN patients and speculated that AN patients rely more on incorrectly perceived proprioceptive information, causing AN patients to misperceive their own body size. If this were the case, we should expect to see a negative correlation between the SWI and visual body size estimates in AN patients, which we did not find. The absence of a correlation should be interpreted with caution but might be indicative of a dissociation of body size and object size processing. Previous research comparing visual body size estimation tasks in AN with neutral object estimation show only overestimation for their own body and not for neutral objects [28, 29], suggesting separate processing systems. Furthermore BID in AN is mostly seen in salient body parts such as the abdomen and hips [28], indicating an emotional component at play. Since this is not the case for objects, we would not expect to see a difference in object perception.

There are several methodological differences between the current study and that of Case et al. [21]. For example, they asked participants to rate the relative heaviness of a small and large disk that were simultaneously placed on the palm of each hand. Our participants lifted the weights consecutively with one hand, while the weight that was not lifted was occluded from sight. Although these procedural differences are noteworthy, they do not offer an explanation for the different results between studies. A more likely explanation is our sample size, which is considerably larger (*n* = 30 per group) compared to that of Case et al. [21] (*n* = 10 per group).

The present study also offers some interesting additional findings. Results did not reveal evidence of motor adaptation in grip force rate for the haptic condition. This could be explained by our finding of a weaker haptic compared to visual SWI. Visual cues are more informative in making size judgements than haptic cues alone [30]. This might cause participants to overestimate the weight of the larger object to a larger extent in the visual compared to the haptic condition. This overestimation of weight might lead to a more rapid scaling of fingertip force to actual object weight in the visual condition, compared to the haptic condition.

Previous studies that investigated the effects of touch and vision on the SWI found an equally strong visual and haptic SWI [31, 32]. In these studies participants were allowed to lift the objects multiple times to compare their weight. In our study participants were only allowed to lift each object once per trial. More experience of same size cues may therefore be necessary in order to elicit a stronger haptic SWI.

The current study did not find a motor adaptation for grip force peak. Motor adaptation for grip force rate is usually seen within the first five lifts [16], perhaps the six lifts used in our study might have been sufficient to show this trend for grip force rate, but still insufficient for peak grip force. The adaptation of grip force rate did not seem to affect perceptual judgements in of the SWI in our sample, supporting the independence of perceptual and sensorimotor predictions of weight [17].

The absence of significant differences in overestimation of width of waist and hips between RAN and HC is in contrast with the findings of our previous study, where we did find an overestimation [8]. In our current study, HC show higher percentages of misestimation compared to our previous study and earlier work on this topic, e.g. [33]. AN and RAN patients show a similar pattern in percentage of overestimation between studies. It might be the case that our HC sample had higher body dissatisfaction rates compared to the sample in our previous study. However, this suggestion should be interpreted with caution since we are unaware of any evidence that body dissatisfaction drives overestimation in body size. Unfortunately, due to time restrictions we did not administer a body satisfaction questionnaire in this study and cannot compare body satisfaction in HC between studies.

In conclusion, we found no differences in the strength of the SWI (visual or haptic) between AN patients, RAN patients and HC, indicating no evidence of visuo-haptic integration deficit. We found that all groups experience a stronger visual than haptic SWI. Results of the grip force rate measurements revealed evidence of motor adaptation for the larger object in the visual modality, but not the haptic modality. Here we suggest that the haptic system needs more (haptic) cues to build up a representation of object size. Furthermore, we did not find a correlation between the VSE and SWI, indicating no relation between estimation of object weight and body size. This is also in line with previous studies showing that object size and body size are processed independently. Together, these results provide no evidence of impaired of object-related multisensory integration in AN.
